# Retraction: Activated α2-macroglobulin binding to cell surface GRP78 induces T-loop phosphorylation of Akt1 by PDK1 in association with raptor

**DOI:** 10.1371/journal.pone.0325677

**Published:** 2025-06-05

**Authors:** The PLOS One Editors

After this article [1] was published, concerns were raised regarding results presented in Figs 1 and 2.

Specifically, the following results appear similar despite representing different experimental conditions:

- Fig 1A IP PDK1 Raptor panel in [1] and Fig 1A EP-2 PC-3 panel in [2, retracted in 3].
- Fig 2A Actin panel in [1], Fig 8C Akt^S473^ panel in [4, retracted in 5], Fig 4A p-Akt^S473^ panel in [6], and Fig 7B p-PRAS panel in [6].
- Fig 2B 1-LN panel in [1] and Fig 10 Insulin dsRictor RNAi p-Akt^S473^ in [4, retracted in 5].
- Fig 2B DU-145 panel in [1] and Fig 6B IB GRP78 panel in [4, retracted in 5].

During editorial follow-up, raw blot data underlying the panels of concern were provided, however these data were insufficient to resolve the concerns listed above and raised further concerns about the labelling, storage, and reliability of the data underlying the results presented in this article.

In light of the above unresolved concerns that question the integrity and reliability of the reported results and conclusions, the *PLOS One* Editors retract this article.

SVP did not respond to the final editorial decision. UKM either did not respond directly or could not be reached.

The Fig 2A Actin panel reports results that appear similar to material from [6] published in 2012 by Wiley, which is not offered under a CC BY license and is therefore excluded from this article’s [1] license.
